# The physical impact of long bone fractures on adults in KwaZulu-Natal

**DOI:** 10.4102/sajp.v76i1.1393

**Published:** 2020-08-20

**Authors:** Sevani Singaram, Mergan Naidoo

**Affiliations:** 1 Department of Public Health Medicine, University of KwaZulu-Natal, Durban, South Africa; 2 Department of Family Medicine, Medical School, University of KwaZulu-Natal, Durban, South Africa

**Keywords:** long bone fracture, physical impact, performing tasks, limitations, KwaZulu-Natal

## Abstract

**Background:**

Limb fractures are increasingly common in low-income and middle-income countries due to an increase in motor vehicle and other accidents. Fractures may often lead to physical impairment that affects an individual’s ability to carry out tasks.

**Objectives:**

To assess the physical impact of long bone fractures on adults in KwaZulu-Natal.

**Method:**

A standardised questionnaire pertaining to activities at home and leisure was used to establish patient-reported outcomes at nine public hospitals. English-speaking and isiZulu-speaking participants who had sustained a single long bone fracture in the preceding 4 to 12 weeks at the time of data collection were included. The following activities were evaluated: walking, running, exercising, driving, performing household chores, writing, answering telephones, texting on a cell phone, bathing, using crockery and preparing meals.

**Results:**

A total of 821 participants completed the questionnaire. Ninety-three per cent had closed long bone fractures and 69 per cent were lower limb fractures. Fifty-seven per cent of the fractures were caused by a fall. Female participants (*p* = 0.19) with lower limb fractures were more likely to have greater difficulty in performing tasks and participants 60 years of age and older (*p* = 0.001) were significantly more likely to have difficulty performing tasks.

**Conclusion:**

These findings illustrate the daily limitations in patients’ everyday activities at home, leisure and in activities such as driving.

**Clinical implications:**

This study highlights the difficulty that some individuals, particularly women and individuals 60 years of age and older, face in performing daily tasks after experiencing a long bone fracture.

## Background

More than half (approximately 57%) of the burden of injuries, which includes injuries due to motor vehicle accidents (MVAs), unintentional injuries and injuries due to violence, are concentrated in low-income and middle-income countries (LMICs) (Prinja et al. 2016). There are approximately 1 million MVAs reported in South Africa alone each year (Daily News Reporter 2018). In particular, KwaZulu-Natal (KZN), which is one of the two most populous provinces in South Africa (Statistics South Africa 2018), recently recorded the highest number of MVAs (Singh & Ndlazi 2019). When motor vehicle accidents occur, long bone fractures are common and often require referral to emergency centres (Frouzan et al. 2017).

The care of patients with fractures also demands an understanding of how the fracture affects their daily activities (Jackson et al. 2014; Modin, Ramos & Stomberg 2009). Preventing and managing these injuries is important for people to perform tasks in the workplace, in the home and participate in sporting and recreational activities (Abernethy & Abernethy 2005). Since the long bones are adapted for the purposes of motion and enable us to shift our position from place to place, long bone fractures often result in a decline in mobility and an inability to perform activities or tasks (Browner et al. 2014; Power 2014; Slobounov 2008).

Specific factors that can affect the extent of physical function after a long bone fracture include age, location of fracture, gender, type of fracture, severity of fracture and time from injury (Mirhadi, Ashwood & Karagkevrekis 2013). Experiencing difficulty in carrying out tasks and relying on others is related not only to a decreased quality of life (QOL), but also an increased likelihood of long-term nursing home placement and dependency on carers, family and friends (Baker et al. 2014; Hamdy 2017). Physical functioning is usually assessed by using specific questions related to mobility status, independence within and outside the home and the ability to perform activities of daily living such as bathing, cleaning and preparing meals.

Many studies (Clement et al. 2014; Jan et al. 2002; Kammerlander et al., 2012; Pils et al. 2013) have assessed the physical impact of vertebral and fragility fractures in the older population. The results reveal that patients with complications such as malunion report greater difficulty in performing tasks, and elderly patients with hip fractures require greater care due to the mortality associated with hip fractures in the elderly. There are very few studies (Singaram & Naidoo, 2019) on long bone fractures in adults across a variety of age groups. Most of the reviewed studies were conducted in high-income and middle-income countries (HMICs) with well-established health systems and most of the participants were elderly (Audibert and Mathonnat 2012; Hohmann, Glatt & Tetsworth 2016). These studies reveal that pain is a common reason for patients to seek medical attention and many patients with lower limb fractures often require hospitalisation.

Our study aimed to assess the physical impact of long bone fractures on adults accessing care in the KZN public healthcare sector. In doing so, our study may inform medical and rehabilitation management and clinical practice by providing cross-sectional data which can assist healthcare professionals in understanding the physical limitations experienced after a long bone fracture across various age groups. To our knowledge, there are no other studies investigating the physical impact of long bone fractures on adults in KZN.

## Methods

Our cross-sectional multi-site study involving patients receiving orthopaedic care at nine KZN public sector hospitals was undertaken to investigate the physical impact of long bone fractures on adults. It was conducted from July 2017 to February 2018.

Only patients 18 years of age and older with one long bone fracture, sustained in the preceding 4 to 12 weeks prior to questionnaire completion, were eligible for inclusion. We chose this time frame because it takes approximately 4 to 12 weeks for a fracture to heal or partially heal, by which time the patients should have been able to truly reflect on the impact of the fracture (Peate et al. 2014). Cognitively impaired participants and those not able to read or write in English or isiZulu were excluded from the study since only English and isiZulu information sheets, consent forms and questionnaires were available. Only participants with fractures of the humerus, radius, ulna, femur, tibia and fibula were included.

The factors that were considered to calculate the sample size were variables of the study, practicality, time frame and cost (Pourhoseingholi et al. 2013). It was assumed that hospitals that belong to the KZN Department of Health provide a service to approximately 50 to 100 patients with long bone fractures per month based on the previous year’s statistics. The sample size was calculated using G*PowerTM. After considering the variables in our study, the sample size was set at 821, with a level of precision of 0.13 at a Type 1 error rate of 5% and a 95% confidence level (Jeovany et al. 2014). A precision of 0.13 yielded a practicable sample size.

To obtain the sample, a two-stage sampling strategy was used. A list of all KZN health institutions was retrieved from the KZN Department of Health website (KwaZulu-Natal Department of Health 2016). In the first stage of sampling, all health institutions that did not provide orthopaedic rehabilitation services or deliver orthopaedic treatment were excluded. In the second stage of sampling, all clinics were excluded and only hospitals with inpatient and outpatient orthopaedic facilities were included. Twenty-seven hospitals were available for inclusion, nine of which granted approval to conduct our study. These nine hospitals are situated in a mix of urban and rural areas and care for both inpatients and outpatients. Owing to the large sample size, cost factors and stringent inclusion and exclusion criteria, convenience sampling was used to select the participants at each hospital.

The number of participants that were selected from each hospital is illustrated in Appendix 2.

### Outcome measure

A questionnaire was utilised to collect information in a standardised format, and it allowed respondents to think carefully about their choices before answering. Further to this, a five-point Likert scale was used to measure the extent to which participants agreed or disagreed with a question.

The self-developed questionnaire consisted of questions to assess the extent of difficulty in performing everyday activities. Some questions were only applicable to those with upper limb fractures or lower limb fractures, while some were applicable to all participants. Our study is part of a larger study that investigated the psychological, social, financial, occupational and physical impact of long bone fractures on adults in KZN. The six variables that were explored using the questionnaire are: psychological impact, social impact, financial situation, occupational impact, physical impact and care and support received from healthcare professionals after a long bone fracture. The sub-variables were: difficulty walking, running, exercising, driving, performing household chores, writing, answering the telephone, texting on a cell phone, bathing, using crockery and preparing meals. The sub-variables were explored in relation to participants’ demographics and injury characteristics.

An extensive literature review was conducted prior to the development of the questionnaire. Content validity is carried out to assess the degree to which an instrument measures each construct. Subject matter experts are usually given the questionnaire or measurement tool to assess how well the questions measure each construct (Bolarinwa 2015). To ensure content validity in our study, the questionnaire and study protocol were sent to 27 medical managers and orthopaedic surgeons, and three provided feedback on the content either telephonically or in writing. The questionnaire was also analysed by members of the relevant institutional research ethics committee who are experienced researchers, and feedback regarding the content of the questionnaire was provided to the authors. After receiving feedback, the first author then engaged with the literature on this topic again to ensure the objectivity of the questions and then made further amendments to the questionnaire. The English questionnaire was translated into isiZulu by a senior academic in the African Languages Department at UKZN.

The accuracy of the questionnaire language was assessed by inviting 20 participants from the pilot study to give feedback on any difficulty experienced when deciphering questions. The research assistants who administered the questionnaire also assessed whether the questionnaire was readable and easy to understand. The internal consistency of the seven-item scale used (Table 4) and the five-item scale used (Table 5) was assessed using Cronbach’s alpha as it is a widely used method for estimating internal consistency or reliability. Both scales demonstrated adequate internal consistency (Oeffinger 2007; Che Rahim & Nasir 2013). Only questions with a high response rate were included in this publication. The questionnaire can be accessed in Appendix 1.

### Data collection

#### Pilot study

The relevant managers and healthcare professionals at the various hospitals were informed about our study after the necessary approvals were granted. To test the data collection process, we conducted a pilot study which included 20 participants from one hospital in July 2017. Data were collected by one research assistant, a homeopath. The research assistant was fluent in English and isiZulu and read and understood the study protocol before data collection. Convenience sampling was used to recruit the 20 participants. Some participants were able to complete the questionnaire on their own while others were assisted by the research assistant. In the latter instance, the research assistant asked the participants the questions and recorded their responses. Each participant spent approximately 30 to 45 minutes completing the questionnaire.

Participant responses were captured onto a database and statistical analysis was performed using Stata 15 by a specialist statistician. Data interpretation was done by both authors to assess if the questionnaire measured the variables and if the aim and objectives of our study were achieved. Participants from the pilot study were not included in the main study and the data from the pilot study were not included in the final analysis. The pilot study was conducted over two days.

#### Main study

Data were collected by two research assistants, a homeopath and a nurse, both of whom had experience in patient care and treatment and were fluent in English and isiZulu. The research assistants spent approximately 10 days collecting data at each hospital. The first author and research assistants were informed on which days to collect data, to ensure there was no interference with medical assessment and treatment of the patients.

Potential participants were recruited with the assistance of orthopaedic staff after reviewing patients’ records and X-rays. Long bone fractures were classified as open and closed and not displaced, moderately displaced and severely displaced after consulting with orthopaedic staff and reviewing patient records and X-rays. The research assistant explained the aim and objectives of our study to the participants and those who wished to be included were given an information sheet to read, and thereafter a consent form to sign. Information sheets, consent forms and questionnaires in isiZulu were given to participants who wished to complete the questionnaire in isiZulu. Some participants were able to complete the questionnaire on their own while those who were unable to write due to the limiting effects of the long bone fracture were assisted by the research assistant. In the latter instance, the research assistant asked the participants the questions and recorded the participants’ responses. Approximately 30 to 45 minutes were spent by each participant on the questionnaire. To limit any bias, the two research assistants were independent practitioners and not employed by the KZN Department of Health, the questions were short and clear, and a simple set of answer options was used.

### Data analysis

Before data capturing, the isiZulu questionnaires were translated back to English by one of the research assistants. The collected data were entered onto a database by a data capturer at the South African Medical Research Council (SAMRC). Ten per cent of the questionnaires were double entered by a second data capturer to ensure quality control. In Table 1, demographic characteristics were summarised as frequencies and percentages. In Tables 2–4, ordinal data were converted to numerical data by assigning numbers to responses on the Likert scale, which were coded as follows: strongly disagree 1, disagree 2, undecided 3, agree 4 and strongly agree 5. Eleven questions were included in the analysis. The Wilcoxon rank sum test was used to determine whether the distribution of responses of self-reported physical disability differed significantly between those with upper and lower limb fractures and between those who were involved in MVAs and those who had falls. Numerous studies have calculated the mean from Likert ordinal scale data (Ahmed Al Kuwaiti 2019; Florin & Sorina 2013; Naidoo 2017).

**TABLE 1:** Demographics and fracture characteristics (*n* = 821).

Demographics	*n*	%
**Age group**		
18–35 years (young)	346	42.14
36–59 years (middle-aged)	319	38.86
60 and over (old)	156	19.00
**Total**	**821**	**100.00**
**Gender**		
Male	519	63.22
Female	302	36.78
**Total**	**821**	**100.00**
**Ethnicity**		
Black	718	87.45
White	19	2.31
Mixed race	3	0.37
Indian	73	8.89
Other	8	0.97
**Total**	**821**	**100.00**
**Patient type**		
Inpatient	645	79.34
Outpatient	168	20.66
Missing responses	8	100.00
**Region of fracture**		
Upper limb	251	30.57
Lower limb	570	69.43
**Fractured long bone**		
Humerus	91	11.08
Radius	86	10.48
Ulna	74	9.01
Femur	310	37.76
Fibula	65	7.92
Tibia	195	23.75
**Severity of fracture**		
Open long bone fracture	57	7.06
Closed long bone fracture	750	92.94
Missing responses	14	-
**Displacement of fracture**		
Severely displaced	148	18.07
Moderately displaced	540	65.93
Not displaced	131	16.00
Missing responses	2	86.71
**Time frame of fracture**		
4–6 weeks	711	86.71
7–8 weeks	60	7.32
9–10 weeks	17	2.07
11–12 weeks	32	3.90
Missing responses	1	-
**Mechanism of injury**		
Motor Vehicle Accident (MVA)	190	23.17
Assault	78	9.51
Sports injury (SI)	17	2.07
Injury due to fall	470	57.32
Other	65	7.93
Missing responses	1	-
**Participants who had surgery**	**161**	**19.73**
Missing responses	5	-
**Employed participants**	**97**	**11.81**

**TABLE 2:** The impact of upper limb fractures on daily functioning (*n* = 251).

Question	Strongly disagree		Disagree		Undecided		Agree		Strongly agree		Missing responses	Total
	*n*	%	*n*	%	*n*	%	*n*	%	*n*	%		
Difficulty walking	N/A	-	N/A	-	N/A	-	N/A	-	N/A	-	N/A	N/A
Difficulty running	N/A	-	N/A	-	N/A	-	N/A	-	N/A	-	N/A	N/A
Difficulty exercising	17	15.60	64	58.72	5	4.59	12	11.01	11	10.09	N/A	109
Difficulty driving	6	11.54	15	28.85	8	15.38	9	17.31	14	26.92	N/A	52
Difficulty performing household chores	5	1.99	64	25.50	11	4.38	109	43.43	62	24.70	N/A	251
Difficulty writing	27	11.20	87	36.10	3	1.24	93	38.59	31	12.86	10	241
Difficulty answering telephone	53	21.9	119	49.17	2	0.83	58	23.97	10	4.13	9	242
Difficulty texting on a cell phone	50	20.66	106	43.80	3	1.24	67	27.69	16	6.61	9	242
Difficulty bathing	3	1.20	53	21.20	2	0.80	139	55.60	53	21.20	1	250
Difficulty handling crockery	16	6.64	111	46.06	2	0.83	94	39.00	18	7.47	10	241
Difficulty preparing meals	16	6.40	95	38.00	8	3.20	102	40.80	29	11.60	1	250

N/A, not applicable.

**TABLE 3:** The impact of lower limb fractures on daily functioning (*n* = 570).

Question	Strongly disagree		Disagree		Undecided		Agree		Strongly agree		Missing responses	Total
	*n*	%	*n*	%	*n*	%	*n*	%	*n*	%		
Difficulty walking	5	0.90	14	2.53	1	0.18	174	31.41	360	64.98	16	554
Difficulty running	3	0.53	12	2.11	7	1.23	170	29.93	376	66.20	2	568
Difficulty exercising	2	0.82	13	5.35	4	1.65	90	37.04	134	55.14	0	243
Difficulty driving	1	0.60	8	4.76	4	2.38	37	22.02	118	70.24	0	168
Difficulty performing household chores	2	0.35	41	7.19	29	5.09	313	54.91	185	32.46	0	570
Difficulty writing	N/A	-	N/A	-	N/A	-	N/A	-	N/A	-	N/A	N/A
Difficulty answering telephone	N/A	-	N/A	-	N/A	-	N/A	-	N/A	-	N/A	N/A
Difficulty texting on a cell phone	N/A	-	N/A	-	N/A	-	N/A	-	N/A	-	N/A	N/A
Difficulty bathing	25	4.39	202	35.50	4	0.70	287	50.44	51	8.96	1	569
Difficulty handling crockery	N/A	-	N/A	-	N/A	-	N/A	-	N/A	-	N/A	N/A
Difficulty preparing meals	73	12.81	294	51.58	26	4.56	155	27.19	22	3.86	0	570

N/A, not applicable.

**TABLE 4:** Pooled analysis – The physical impact of the long bone fracture: Motor vehicle accidents versus falls (*n* = 821).

Question	Mechanism of injury	*n*	Mean	Standard deviation	*p*
Difficulty walking	MVA	141	4.66	0.63	0.16
	Fall	325	4.54	0.77	
Difficulty running	MVA	145	4.65	0.65	0.15
	Fall	334	4.56	0.72	
Difficulty exercising	MVA	78	3.97	1.32	0.22
	Fall	200	3.84	1.25	
Difficulty driving	MVA	67	4.45	0.97	0.20
	Fall	114	4.26	1.07	
Difficulty performing household chores	MVA	190	4.09	0.90	0.21
	Fall	470	3.98	0.96	
Difficulty writing	MVA	44	3.34	1.40	0.09
	Fall	131	2.98	1.24	
Difficulty answering telephone	MVA	44	2.57	1.26	0.27
	Fall	130	2.33	1.12	
Difficulty texting on a cell phone	MVA	44	2.68	1.34	0.58
	Fall	130	2.55	1.23	
Difficulty bathing	MVA	190	3.38	1.19	0.92
	Fall	468	3.42	1.11	
Difficulty handling crockery	MVA	44	3.18	1.24	0.15
	Fall	129	2.88	1.12	
Difficulty preparing meals	MVA	190	2.86	1.22	0.17
	Fall	469	2.71	1.16	

MVA, Motor vehicle accident.

In Table 5 and Table 6, an analysis of variance (ANOVA) was used to test for statistically significant differences in physical disability scores between age groups and gender in those with upper limb and lower limb fractures. To obtain the scores for upper limb fractures, questions with a response rate of 80% or more were included in the analysis. The questions included addressed: difficulty performing household chores, difficulty writing, difficulty answering telephones, difficulty texting on a cell phone, difficulty bathing, difficulty handling crockery and difficulty preparing meals. Since seven categories were analysed, and the highest numerical code assigned to a response was 5, the overall score was out of 35. To obtain the scores for lower limb fractures, questions with a response rate of 80% or more were included in the analysis. The questions included addressed: difficulty walking, difficulty running, difficulty performing household chores, difficulty bathing and difficulty preparing meals. The mean scores were categorised under two groups of interest, age and gender. Since five categories were analysed and the highest numerical code assigned to a response was 5, the maximum score was out of 25.

**TABLE 5:** Disability scores for physical impact of upper limb fractures (*n* = 234).

Category	*n*	Mean	Standard deviation	*p*
**Age**				**0.54**
18–35	111	21.24	6.16	
36–59	94	21.45	6.05	
60+	29	22.66	5.79	
Total	234	21.50	6.06	
**Gender**				**0.49**
Male	157	21.30	6.26	
Female	77	21.90	5.65	
Total	234	21.50	6.06	

**TABLE 6:** Disability scores for physical impact of lower limb fractures (*n* = 551).

Category	*n*	Mean	Standard deviation	*p*
**Age**				0.001*
18–35	215	18.46	2.93	
36–59	213	19.05	2.57	
60+	123	20.20	2.60	
Total	551	19.07	2.79	
**Gender**				**0.19**
Male	340	18.95	2.73	
Female	211	19.27	2.88	
Total	551	19.07	2.79	

* , statistical significance

A Tukey post hoc test was used for pairwise comparisons between age groups. All analyses were performed using the statistical software Stata 15; *p* values less than 0.05 were considered statistically significant.

### Ethical consideration

Ethical clearance was sought and obtained from the University of KwaZulu-Natal Biomedical Research Ethics Committee, reference number: BE583/16.

## Results

The participants’ demographics and fracture characteristics are summarised in Table 1.

Most participants were younger than 60, with 346 (42.14%) and 319 (38.86%) aged 18–35 and 36–59.

Approximately two-thirds of the participants were male, 519 (63.22%). Most fractures were closed long bone fractures (92.94%). A higher proportion of participants sustained fractures to the lower extremity, 570 (69.43%).

The long bone most fractured was the femur, 310 (37.76%) and the least fractured bone was the fibula, 65 (7.92%). The most common mechanism of injury was falls. The second most common mechanism of injury was MVAs.

Table 2 demonstrates the impact of upper limb fractures on daily functioning. The task that participants had the most difficulty performing was bathing.

Table 3 shows the impact of lower limb fractures on daily functioning. Participants reported the greatest difficulty walking. There were statistically significant differences (*p* value 0.001) between upper and lower limb fractures for the following activities: exercising, driving, performing household chores, bathing and preparing meals, with lower limb fracture participants having more difficulty with exercising, driving, doing chores and preparing meals. Participants with upper limb fractures had more difficult bathing.

The two commonest mechanisms of injury, falls and MVAs, were compared with the disability experienced and this is tabulated in Table 4.

Table 4 demonstrates that those who had MVAs had greater difficulty in performing most tasks compared to those who fell. There were no significant differences in disability scores.

Table 5 shows the scores for the physical impact of upper limb fractures, which demonstrates the level of difficulty participants with upper limb fractures had when categorised by age and gender. Females and those who were 60 years and older had higher mean scores, indicating that they had greater difficulty performing tasks. Pairwise comparisons between the 36–59 versus 18–35 year age group, 60+ versus 18–35 year age group, and 60+ versus 36–59 year age group revealed no significant differences in scores. The results of the pairwise comparisons are demonstrated in Appendix 3.

Table 6 shows the scores for the physical impact of lower limb fractures, which demonstrates the level of difficulty participants with lower limb fractures had when categorised by age and gender. Females and those who were 60 years and older had higher mean scores, indicating that they had greater difficulty in performing tasks. There was a statistically significant difference in pairwise comparisons between the 60+ and 18–35 year age group (*p* value 0.001) and between the 60+ and 36–59 year age group (*p* value 0.001). The results of the pairwise comparisons are shown in Appendix D.

## Discussion

Our study assessed the physical impact of long bone fractures on adults in KZN. Eight hundred and twenty-one participants with long bone fractures were included in the final analysis. Most participants had lower limb fractures; they were younger than 60 and approximately two-thirds of the sample was male. Most fractures were closed and occurred as a result of falls. Participants with lower limb fractures had more difficulty with most daily tasks compared to those with upper limb fractures. There were no differences in age and gender in the difficulty performing tasks in participants with upper limb fractures; however, those with lower limb fractures who were over 60 years of age had more difficulty performing tasks. Women with lower limb fractures also had more difficulty performing tasks, although this was a non-significant difference.

The two tasks that those with lower limb fractures had the most difficulty performing were walking and running. A decline in locomotor function due to a fracture can lead to a decline in habitual walking, reductions in gait stability and efficiency in walking, alterations to the gait kinematics and alterations to the kinetics of walking (Mian et al. 2007). A lack of ability to walk places the patient at a greater risk for skin ulcers, deep vein thrombosis and pneumonia (Eiff et al. 2018; Fulde 2004; Luqmani 2013). Unintentional weight gain after a lower limb fracture is due to limited ability to move because of pain or immobilisation (McPhail et al. 2012). Our study demonstrated that those with lower limb fractures had greater difficulty exercising compared to those with upper limb fractures.

Holtslag and colleagues (Holtslag et al. 2006) investigated the impact of lower limb injuries on physical activity and found that 60% described that they were limited in walking. Their prospective cohort study set in Europe included 186 participants (mean age 37 years; 139 male, 47 female) with lower extremity injuries, 80% of which had resulted from a MVA. McPhail et al. (2012) in a qualitative investigation of the impact of distal tibia or fibula fractures found personal care tasks were impacted. In an Australian study, most patients described how pain or swelling limited their ability to walk and more than half also had difficulty with a personal care task, such as bathing. The difficulty of the movement also depends on the external environment within which it is occurring, the space within which to move and muscle strength (Everett & Kell 2010).

Our study demonstrated that participants with upper limb fractures had the most difficulty with bathing. Fractures to the upper limb can cause significant difficulties in carrying out activities of personal hygiene and consuming food and drink particularly if the dominant side is injured. A common complication that is often associated with neglecting regular personal hygiene is dermatitis neglecta, due to a build-up of sweat, sebum and debris due to inadequate cleaning of the skin (Langar & Sonthalia, 2018; Qadir et al. 2008).

Older patients with lower limb fractures had more difficulty with tasks. Age-related changes may affect the inflammatory response after a fracture. Since the inflammatory response is responsible for debriding the fracture site, this may affect fracture healing (Orive et al., 2015; Power 2014). Elderly frail people may need greater support in coping with fractures, especially during the period of immobilisation (Eiff et al. 2018, Fulde 2004). Swelling is a common complication after a fracture and should be appropriately managed with both medical and physiotherapy guidance (Cheing, Wan & Kai 2005; Power 2014). Complications of fractures to the upper limb include neurovascular compromise, damage to tendons and compartment syndrome. Late complications include carpal tunnel syndrome, malunion, post traumatic arthritis and residual stiffness of the elbow, wrists and fingers (Power 2014).

There were no differences between the ability of men and women with upper limb fractures to perform tasks but there was a non-significant increase in women with lower limb fractures experiencing difficulty in performing tasks. Kempen and colleagues (Kempen et al. 2003) found that after one year, female patients did not recover the ability to perform basic activities compared to men. Age and gender differences appear to affect recovery from a fracture and could be due to muscle strength differences (Schultz et al. 1997)

Driving is a crucial indicator of independence, not only as a means of transportation, but also as a facilitator of independence. In our study a high percentage of those with lower limb fractures had difficulty driving. Lower limb fractures often result in prolonged reductions in mobility (Dischinger et al. 2004). In their Swedish study, Modin et al. (2009) noted that patients with lower extremity fractures found coping with household chores the most difficult. The dominating cause of the limitation was having to use crutches, which meant that both hands were occupied. They also found that many participants were unable to drive and feared walking; both factors that limited their social activities. Similarly, in our study those with lower extremity fractures had greater difficulty in performing household chores compared to those with upper extremity fractures.

Falls and MVAs were the two commonest mechanisms of injury. Participants who had MVAs had higher disability scores for 10 out of 11 activities compared to those who had falls. MVAs are considered high-energy injuries whereas falls are low-energy injuries (Levine et al. 2013; McRae, Esser & Esser 2008). Life-threatening injuries such as a haemorrhage may accompany high-energy injuries (Birrer & Kalb 2015). Low-energy injuries result from minimal trauma and while most low-energy fractures can be treated conservatively, high-energy fractures are usually associated with multi-system morbidity (Asensio & Trunkey 2016). Insufficient sleep duration, alcohol consumption and history of past fracture are usually associated with low-energy fractures (Zhu et al. 2019). The course of healing for low-energy injuries is usually minor whereas the course of healing for high-energy fractures may be complex as surgery may often be required (Cohen et al. 2016) Therefore, the rehabilitative treatment planning for low-energy and high-energy fractures may differ.

Rehabilitative programmes play an important role in the care of patients after a fracture, especially if they include interventions such as technology, psychosocial support and nutritional assistance, as they allow for maximum recovery and may prevent falls and consequently further fractures (Alexiou et al., 2018; Perracini et al. 2018). Rehabilitation programmes should therefore have a major psychosocial component and include a multidisciplinary team such as physiotherapists, orthopaedic surgeons, nurses and psychologists, with a focus on physical and mental well-being of patients (Aylott et al. 2019; Stenvall et al. 2007). Pol et al. (2019a, 2019b) recommend the use of technology-enhanced rehabilitation to encourage older adults to become more active and engaged in the fracture recovery process. Sensor monitoring provides feedback to participants, who become aware of their movements after a fracture, and this allows them to self-correct based on their rehabilitation plan.

## Limitations

Due to the nature of our study, causal relationships between variables cannot be identified. The authors therefore only commented on associations between variables. Self-reported questionnaires are also dependent on the question interpretation of the participants, which may affect the results.

## Conclusion

Our study contributes cross-sectional data to improve our knowledge on the physical impact of long bone fractures in KZN adults, particularly how the fracture affects daily activities. When planning rehabilitation programmes for patients with long bone fractures to the upper and lower extremity, healthcare professionals should consider that female participants and those aged 60 and over may require a longer time to recover.

## Appendix 1: Questionnaire

### Participant number: Please tick the answer that is most appropriate.

#### 1. Demographic Questions

1.1. What is your gender?
  **Table:**
  Male	Female
1.2. What is your race?
  **Table:**
  Black	White	Coloured	Indian	Other
1.3. What is your age?
  **Table:**
1.4. I was treated as an:
  **Table:**
  Inpatient	Outpatient
1.5. Type of fracture:
  - **Table:**
    Upper Limb:	List Bone: humerus/ulna/radius
    Lower Limb:	List Bone: femur/tibia/fibula
  - **Table:**
    Open	Closed
  - **Table:**
    Severely Displaced
    Moderately Displaced
    Not Displaced
1.6. How many weeks ago did the injury take place?
  **Table:**
  4 weeks	5 weeks	6 weeks	7 weeks	8 weeks	9 weeks	10 weeks	11 weeks	12 weeks
1.7. How did injury occur?
  **Table:**
  Motor vehicle accident	Assault	Sports Injury	Injury due to a fall	Gunshot	Other (Please specify)
1.8. Was surgery performed?
  **Table:**
  Yes	No
1.9. If Yes, Please indicate what type of surgery was performed?
  **Table:**

#### After sustaining the fracture:

1. I was bound to a wheelchair
  **Table:**
  Yes	No	N/A- Upper Limb fracture
2. I had to use crutches or a plaster of Paris cast
  **Table:**
  Yes	No
3. I had difficulty walking
  **Table:**
  Strongly Disagree	Disagree	Undecided	Agree	Strongly agree	N/A – Upper Limb Fracture
4. I had difficulty running
  **Table:**
  Strongly Disagree	Disagree	Undecided	Agree	Strongly agree
5. I had difficulty exercising
  **Table:**
  Strongly Disagree	Disagree	Undecided	Agree	Strongly agree	I do not exercise
6. I had difficulty driving
  **Table:**
  Strongly Disagree	Disagree	Undecided	Agree	Strongly agree	I do not drive a motor vehicle
7. I had difficulty performing household chores
  **Table:**
  Strongly Disagree	Disagree	Undecided	Agree	Strongly agree
8. I had difficulty performing tasks at work
  **Table:**
  Strongly Disagree	Disagree	Undecided	Agree	Strongly agree	N/A
9. I had difficulty writing
  **Table:**
  Strongly Disagree	Disagree	Undecided	Agree	Strongly agree	N/A- Lower Limb Fracture
10. I had difficulty answering telephones
  **Table:**
  Strongly Disagree	Disagree	Undecided	Agree	Strongly agree	N/A- Lower Limb Fracture
11. I had difficulty texting on a cell phone
  **Table:**
  Strongly Disagree	Disagree	Undecided	Agree	Strongly agree	N/A- Lower Limb Fracture
12. I had difficulty using the computer or laptop
  **Table:**
  Strongly Disagree	Disagree	Undecided	Agree	Strongly Agree	I did not use a computer or laptop	N/A- Lower limb fracture
13. I had difficulty bathing
  **Table:**
  Strongly Disagree	Disagree	Undecided	Agree	Strongly agree
14. I had difficulty handling crockery
  **Table:**
  Strongly Disagree	Disagree	Undecided	Agree	Strongly agree	N/A- Lower Limb Fracture
15. I had difficulty preparing meals
  **Table:**
  Strongly Disagree	Disagree	Undecided	Agree	Strongly agree

## Appendix 2: Number of participants chosen from each hospital.

**Table:**

Hospital	Frequency	Per cent
A	204	24.9
B	112	13.6
C	155	18.9
D	60	7.3
E	111	13.5
F	121	14.7
G	21	2.6
H	5	0.6
I	32	3.9

## Appendix 3: Pairwise comparisons between age groups: Upper limb fractures

**Table:**

Age category	*p* value (*p* > [t])
36–59 vs 18–35	0.97
60+ vs 18–35	0.51
60+ vs 36–59	0.62

## Appendix D: Pairwise comparisons between age groups: Lower limb fractures

**Table:**

Age category	*p* value (*p* > [t])
36–59 vs 18–35	0.06
60+ vs 18–35	0.00*
60+ vs 36–59	0.00*

* , statistical significance.
